# Base Editing of Human Pluripotent Stem Cells for Modeling Long QT Syndrome

**DOI:** 10.1007/s12015-021-10324-6

**Published:** 2022-01-08

**Authors:** Fujian Wu, Tianwei Guo, Lixiang Sun, Furong Li, Xiaofei Yang

**Affiliations:** 1 Translational Medicine Collaborative Innovation Center, The Second Clinical Medical College (Shenzhen People’s Hospital), Jinan University, Shenzhen, 518020 China; 2 Post-doctoral Scientific Research Station of Basic Medicine, Jinan University, Guangzhou, 510632 China; 3 Guangdong Engineering Technology Research Center of Stem Cell and Cell Therapy, Shenzhen, 518020 China; 4 Shenzhen Key Laboratory of Stem Cell Research and Clinical Transformation, Shenzhen, 518020 China; 5 Beijing Anzhen Hospital, Capital Medical University, Beijing, China; 6 Department of Cardiology, The First Affiliated Hospital of Guangxi Medical University & Guangxi Key Laboratory of Precision Medicine in Cardio-cerebrovascular Diseases Control and Prevention & Guangxi Clinical Research Center for Cardio-cerebrovascular Diseases, Nanning, China

**Keywords:** Base editing, hPSCs, Point mutation, Disease model, LQT

## Abstract

Human pluripotent stem cells (hPSCs) have great potential for disease modeling, drug discovery, and regenerative medicine as they can differentiate into many different functional cell types via directed differentiation. However, the application of disease modeling is limited due to a time-consuming and labor-intensive process of introducing known pathogenic mutations into hPSCs. Base editing is a newly developed technology that enables the facile introduction of point mutations into specific loci within the genome of living cells without unwanted genome injured. We describe an optimized stepwise protocol to introduce disease-specific mutations of long QT syndrome (LQTs) into hPSCs. We highlight technical issues, especially those associated with introducing a point mutation to obtain isogenic hPSCs without inserting any resistance cassette and reproducible cardiomyocyte differentiation. Based on the protocol, we succeeded in getting hPSCs carrying LQTs pathogenic mutation with excellent efficiency (31.7% of heterozygous clones, 9.1% of homozygous clones) in less than 20 days. In addition, we also provide protocols to analyze electrophysiological of hPSC-derived cardiomyocytes using multi-electrode arrays. This protocol is also applicable to introduce other disease-specific mutations into hPSCs.

**Graphical abstract:**

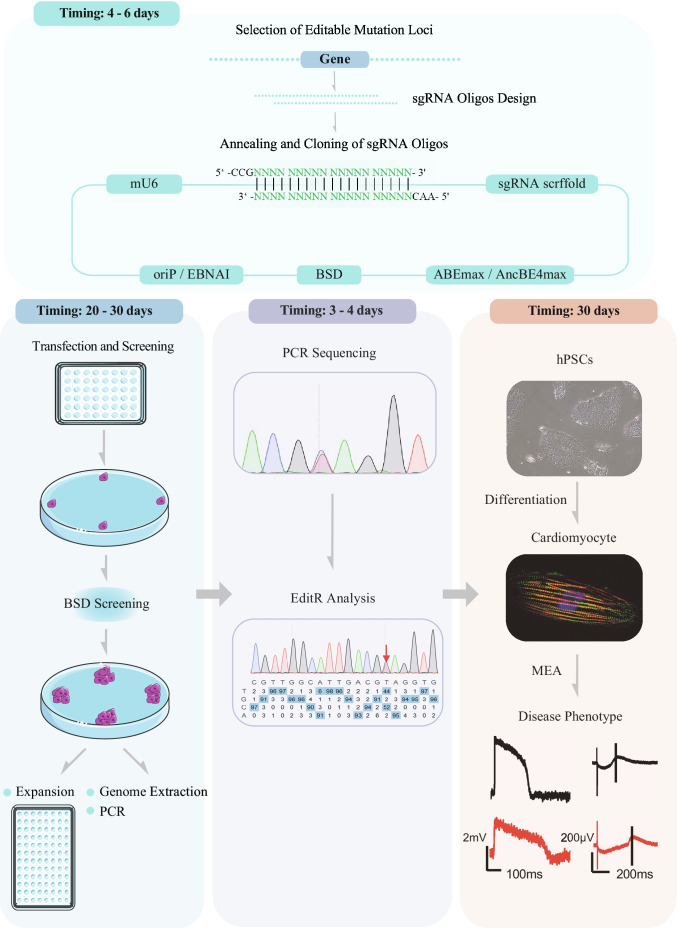

## Introduction

Human pluripotent stem cells (hPSCs), including human embryonic stem cells (hESCs) and the closely related human induced pluripotent stem cells (iPSCs), are characterized by self-renewal and can differentiate into a huge number of different functional cell types via directed differentiation [1]. The ability to proliferate indefinitely allows large number of differentiated derived cells to be obtained in a short period. It plays a vital role in regenerative medicine. The ability to directionally differentiate into somatic cells allows stem cells to play an essential role in disease models [2], drug screening [3], cell development [4], and cell fate choice [5]. Patient tissue-derived iPSCs [6], which are then differentiated into cardiac [7], neural [8], endothelial [9], and other cells, are widely used in disease modeling. Using patient-derived iPSCs, we can study many genetic diseases, such as long QT syndrome [10], Brugada syndrome [11], hypertrophic cardiomyopathy [12], etc. However, some diseases are not genetically inherited [13], and such diseases-derived iPSCs are likely to be non-phenotype observed. For example, some diseases in which methylation is involved in regulation may be lost during reprogramming [14]. In addition, there is a lack of ideal control when compared with patient-derived iPSCs. Researchers usually select healthy people of the same family [15] or unrelated healthy people [16] as control. This may considerably reduce the reliability of the studies, as the genetic backgrounds of these individual are different. Moreover, the reprogramming process from tissue cells to iPSCs is time-consuming [17]. Using gene-editing technology to introduce disease mutations in hPSCs and using unedited hPSCs as control can be a perfect solution to overcome these limitations.

Base editing technique is an evolution of the Clustered Regularly Interspaced Short Palindromic Repeat (CRISPR) system, introducing point mutations without requiring DNA double-strand breaks or donor templates [18]. Under the guidance of sgRNA, the catalytically impaired Cas9 protein fused with a single-stranded DNA deaminase enzyme is guided to the target sequence and then substituted the bases [19]. Because it consists of inactivated Cas9, which undergoes point substitution without producing double-stranded DNA breaks, the non-specific activity is greatly reduced and is considered to be the safest. Three main classes of base editors have been developed to date: cytosine base editors (CBEs), which catalyze the conversion of C•G base pairs to T•A base pairs [20]; and adenine base editors (ABEs), which catalyze A•T-to-G•C conversions [21]; Glycosylase base editors (GBEs), which catalyze the conversion of C•G base pairs to A•G (in bacteria) [22] and catalyze the conversion of C•G base pairs to G•A (in mammalian cells) [23]. These techniques could theoretically be used to correct or introduce human pathogenic SNPs [24]. Conventional base editing techniques require two vectors. One expresses catalytically impaired Cas9 protein fused with a single-stranded DNA deaminase enzyme, the other expressing sgRNA. Gene editing is only possible happened if two plasmids enter a cell at the same time. However, although the efficiency of gene editing is high, the low efficiency of hPSCs transfection is a huge obstacle [25]. Therefore, the introduction of disease mutations in hPSCs is a very time-consuming and challenging work, but it is easier and shorter than reprogramming.

**Fig. 1:**
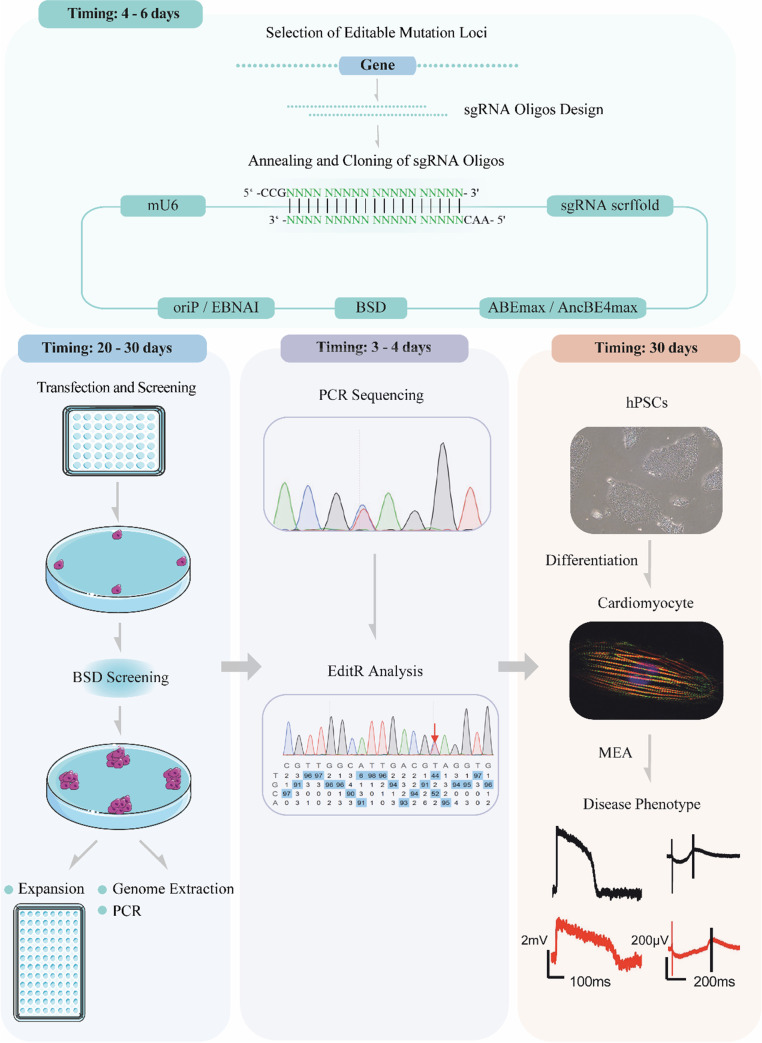
Overview of base editing of hPSCs for modeling LQT syndrome

**Fig. 2:**
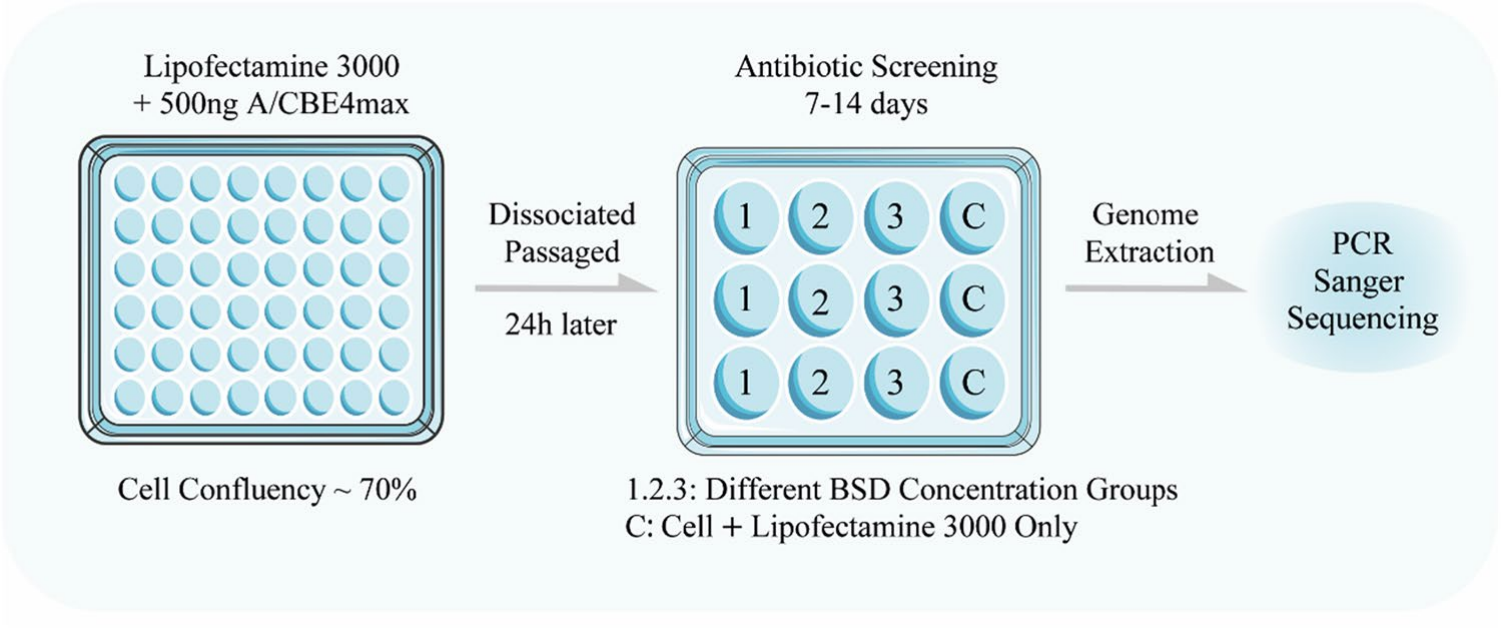
Schematic diagram of cell transfection and antibiotic screening

**Fig. 3:**
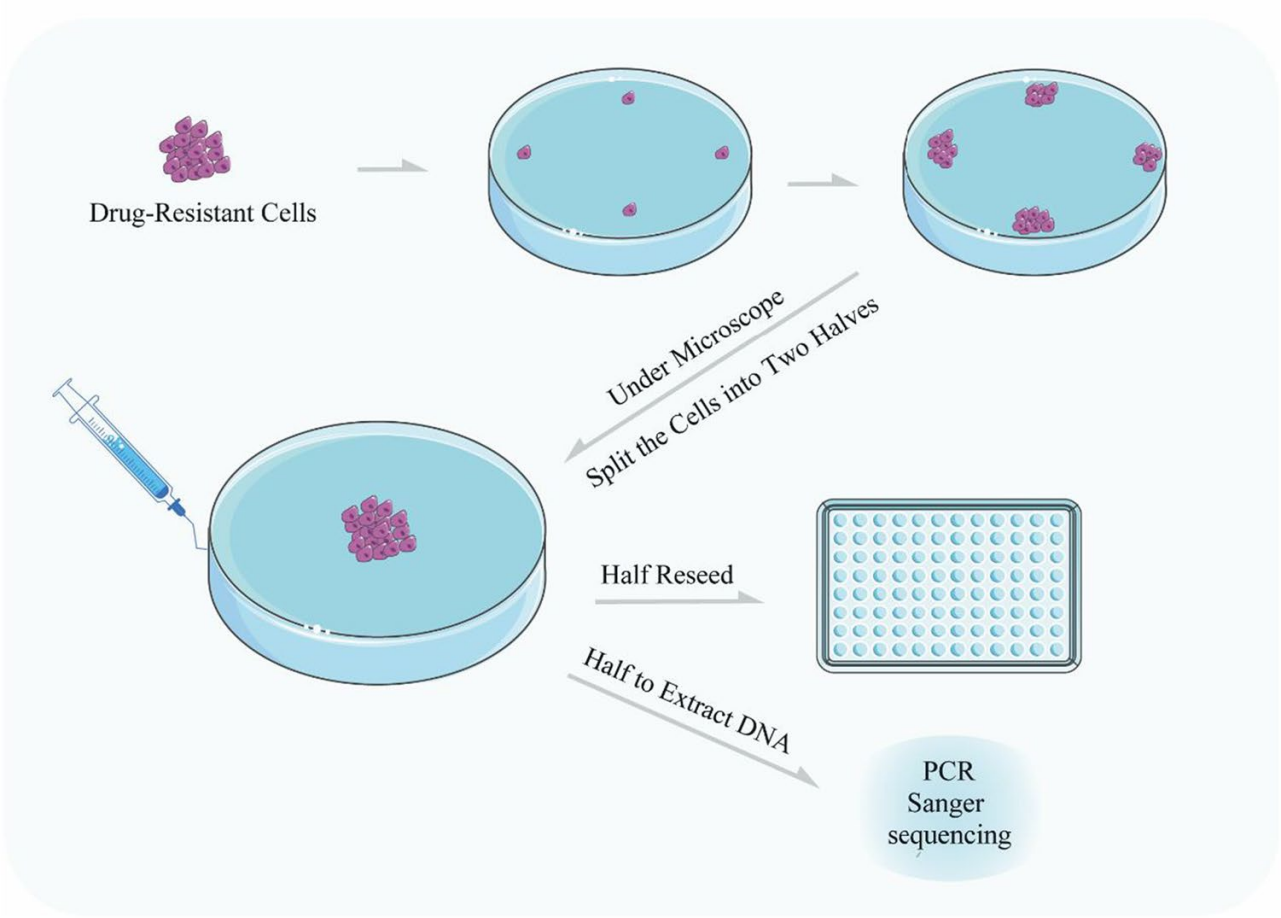
Schematic diagram of single cell clone screening

**Fig. 4:**
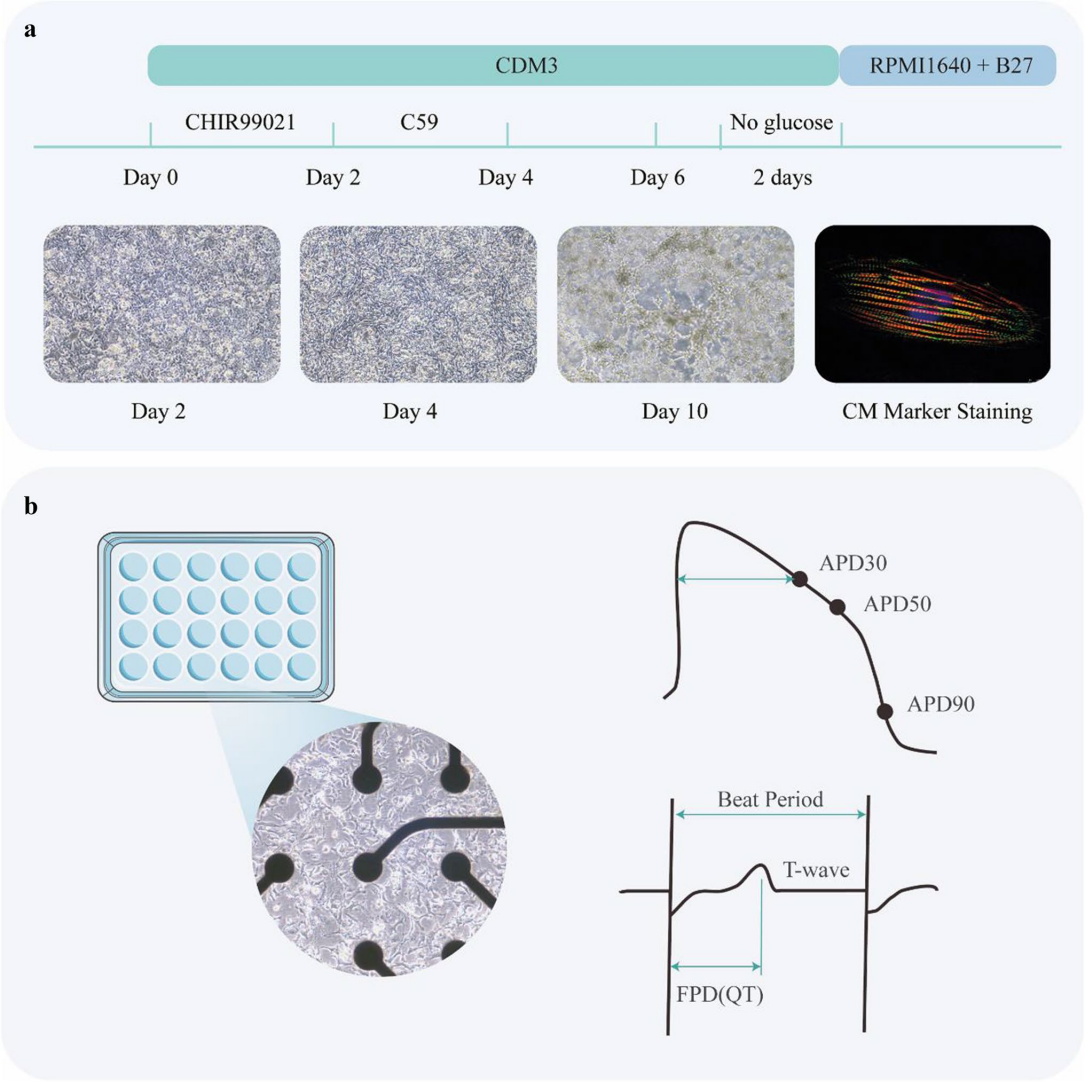
Cardiomyocytes differentiation and MEA testing. **a** Schematic diagram of the process of cardiomyocytes differentiation and photographs of cells at different days. **b** Cardiomyocytes plated on MEA plates and diagram of MEA results analysis

In our previous work [26], inactivated Cas9, a single guide RNA (sgRNA) with an adenine base editor (ABE) or a cytosine base editor (CBE), were all co-expressed in one episomal vector (refer to epi-BE). The episomal vector can replicate during cellular division in eukaryotes permitting the continuous expression of Cas9, base editor, and sgRNAs in hPSCs. epi-BE also contains a drug resistance gene. Despite the low efficiency of plasmid delivery, we were able to greatly enrich the target cells through long-term drug screening and the proliferation of drug-resistant cells. We introduced mutations in three pathogenic genes of LQT, KCNQ1, KCNH2, and SCN5A, and screened a total of 328 clones, of which 104 were heterozygous (31.7%) and 30 were homozygous (9.1%) (Note 12). In this paper, we will show how to introduce LQT disease mutation loci into hPSCs step by step. To model LQT syndrome, the diseased hPSCs are differentiated into cardiomyocytes for phenotypic and functional characterization Figs. 1, 2, 3 and 4.

## Materials

### Reagents

#### Design of the Plasmids for Base Editing and Functional Analysis

- Plasmids: epi-ABEmax (Addgene plasmid #135974), epi-BE4max (Addgene plasmid #135975).
- PCR primers or oligos for sgRNA construction can be ordered with standard desalting purification at GUANGZHOU IGE BIOTECHNOLOGY LTD or other suppliers.
- PrimeSTAR® Max DNA Polymerase (TAKARA, cat. no. R045Q).
- FastPure® Gel DNA Extraction Mini Kit (Vazyme, cat. no. DC301)
- EndoFree Mini Plasmid Kit II (TIANGEN, cat.no. 4992422)
- Agarose (Sigma, cat. no. A9539)
- 100 bp DNA Ladder (Vazyme, cat.no. MD104-01)
- Ultra GelRed Nucleic Acid Stain (Vazyme, cat. no.GR501-01)
- BspQI (New England BioLabs, cat. no. R0712S)
- T4 DNA Ligase (Vazyme, cat. no. C301-01)
- FastPure Plasmid Mini Kit (Vazyme, cat. no. DC201-01)
- DH5α chemically competent *E. coli* (Vazyme, cat. no. C502-02)
- Ampicillin, 100 mg/ml (Beyotime, cat. no. ST008)
- TIANamp Genomic DNA Kit (TIANGEN, cat.no. 4992254)

#### Cell Culture and Cardiomyocytes Differentiation

- HEK293T cell line (Life Technologies, cat. no. R70007)
- Human Embryonic Stem Cell H9 (National Collection of Authenticated Cell Cultures, China, cat. no. SCSP-302)
- DMEM, high glucose (Life Technologies, cat. no. 10566016)
- Dulbecco’s PBS w/o Ca2+, Mg2+ (D-PBS) (Hyclone, cat. no. SH30028.02)
- Fetal Bovine Serum (FBS) (Life Technologies, cat. no.10270-106)
- Opti-MEM™ reduced-serum medium (Life Technologies, cat. no. 11058-021)
- Penicillin/streptomycin (Pen/strep), 100× (Life Technologies, cat. no. 15140-122)
- EDTA (Cellapy, cat. no.CA3001500)
- Lipofectamine 3000 transfection reagent (Life Technologies, cat. no. L3000008)
- Accutase® (Sigma, cat. no. A6964-500ML)
- ROCK1 inhibitor (Y-27632) (Selleck, cat. no. S6390)
- P3 primary cell 4D-Nucleofector X kit S (Lonza, cat. no. V4XP-3032)
- L-ascorbic acid 2-phosphate (Sigma, cat. no.49752)
- Recombinant Human Serum Albumin (Science Cell, cat. no. OsrHSA)
- B-27™ Supplement, minus insulin (Life Technologies, cat. no. A1895601)
- B-27™ Supplement, serum free (Life Technologies, cat. no. 17504044)
- RPMI 1640 Medium (Life Technologies, cat. no. 61870150)
- RPMI 1640 Medium, no glucose (Life Technologies, cat. no. 11879020)
- CHIR-99021(Selleck, cat. no. S1263)
- Wnt-C59 (Selleck, cat. no. S7037)
- mTeSR-1 medium (STEMCELL Technologies, cat. no. 85850)
- Matrigel® hESC-Qualified Matrix (Corning, cat. no. 354277)
- Sodium DL-lactate (Sigma, cat. no. 71720)
- Blasticidin S HCl (Selleck. cat. no. S7419)

### Equipment

- Standard microcentrifuge tubes, 1.5 ml (Eppendorf, cat.no. 0030125150)
- 15 ml Centrifuge Tube (Corning, cat. no. 430791)
- Tissue culture dish, 100 × 20 mm (Corning, cat. no. 353003)
- Tissue culture plate, 6 wells (Corning, cat. no. 353934)
- Tissue culture plate, 96 wells (Corning, cat. no. 353075)
- Cellometer AUTO T4 Bright Field Cell Counter (Nexcelom, cat. no. AUTO T4)
- NanoDrop 2000 device, UV spectrophotometer (ThermoScientific)
- 4D-Nucleofector™ System (Lonza, cat. no. AAF-1002Band AAF-1002X)
- 1 ml sterile syringe (WEIGAO Group Medical Polymer CO., LTD, cat. no. ZSQWG1)

### Softwares and Online Tools

- CRISPR/Cas plasmids and resources on Addgene at https://www.addgene.org/CRISPR/
- BLAST, human genome online tool: http://blast.ncbi.nlm.nih.gov/Blast.cgi
- BASE EDITING ANALYSIS TOOL: https://hanlab.cc/beat/
- ImageJ, quantification software available at http://rsbweb.nih.gov/ij/
- Snapgene: https://www.snapgene.com
- Primer designing tool: https://www.ncbi.nlm.nih.gov/tools/primer-blast/
- EditR: http://baseeditr.com/
- Igor Pro: https://www.wavemetrics.com
- AxIS Navigator, Cardiac Analysis Tool: https://www.axionbiosystems.com/products/software
- Cas-Offinder: http://www.rgenome.net/cas-offinder/
- HGMD®: http://www.hgmd.cf.ac.uk/ac/index.php
- BE-Hive: https://www.crisprbehive.design

### Culture Medium

- HEK293T cell culture medium (500 ml): 440 ml DMEM, 50 ml FBS, 5 ml Pen/Strep. Store at 4 °C.
- CDM3 (500 ml): 500 ml RPMI 1640, 0.25 g of Recombinant Human Serum Albumin, and 106.5 mg of L-ascorbic acid 2-phosphate. Store at 4 °C.
- Collagenase solution (2 mg/mL): Dissolve 500 mg of collagenase type I in 200 mL of D-PBS. Add 50 mL of fetal bovine serum

## Methods

### Selecting Editable LQT Disease Mutation Loci

- The widely used base editing tools ABE or CBE can replace A with G and C with T. Based on this, our choice of mutation sites for LQT disease should be C > T, or A > G (for antisense chains, it is G > A, T > C). More than 90% of LQT is caused by mutations in the *KCNQ1*, *KCNH2*, and *SCN5A* genes. Therefore, we mainly screened for these three genes. The Human Gene Mutation Database (HGMD®) is a robust database that collates all known (published) gene lesions responsible for human inherited disease. We use this database to screen LQT mutations.
- Input *KCNQ1* OR *KCNH2* OR *SCN5A*
- Select: Gene Symbol
- Select: Go!
- Select: Missense/nonsense
- Click: Get mutations
- Check: “codon change” and “Phenotype”
- All A > G, G > A, C > T, T > C mutation loci were recorded
- Open the NCBI gene database https://www.ncbi.nlm.nih.gov/gene/?term=
- Enter the gene name (for example: *KCNQ1*)
- Click “Search”
- Chose “*Homo sapiens*”
- Click “GenBank”
- Click “Send to”
- Download the gene sequence file

(See Note 1)

### sgRNA Oligos Design

After the sgRNA is designed, we can first evaluate its editing efficiency on the BE-Hive website. To evaluate sgRNAs, on the website:

- Step1: Input Sequence: paste your target sequence into the input sequence box
- Step2: select: base editor/cell type
- Step3: chose CRISPR protospacer

### Annealing and Cloning of sgRNA Oligos

1. Pick a 20 bp spacer ahead of the PAM sequence (5’-NGG-3′) in the target locus, and then synthesize the two oligonucleotides as follows:
  Top: 5’-**ttt**NNNNNNNNNNNNNNNNNNNN-3’.
  Bottom: 5’-**aac**N’N’N’N’N’N’N’N’N’N’N’N’N’N’N’N’N’N’N’N′-3’.
  For example, the selected spacer and PAM sequence for the KCNQ1^L114P/+^ gene is 5’-GCTCGAGGAAGTTGTAGACG-CGG −3′. The sequences of KCNQ1^L114P/+^ sgRNA oligonucleotides are as follows:
  KCNQ1^L114P/+^ Top: 5’-**ttt**GCTCGAGGAAGTTGTAGACG -3’.
  KCNQ1^L114P/+^Bottom: 5’-**aac**CGTCTACAACTTCCTCGAGC -3′.
  (See Note 2)
2. Annealing the oligonucleotides as indicated below.

**Table:** 

100uM KCNQ1^L114P/+^ Top	1ul
100uM KCNQ1^L114P/+^ Bottom	1ul
10X T4 DNA ligation buffer	1ul
ddH_2_O	7ul

Place the above samples in a 95 °C water bath, switch off the power, and cool naturally to room temperature. *Alternatives:* You can anneal the oligonucleotides using a thermocycler instead of a hot water bath. Incubate the reaction solution at 95 °C for 5 min and then slowly cool it down to room temperature (20-30 °C) using a thermocycler—the temperature decreases by 1 °C per 10s.

3. Dilute the annealed oligonucleotides 20 folds with ddH2O. Clone the annealed oligonucleotide into the sgRNA expression plasmid as indicated below.

**Table:** 

Diluted annealed oligonucleotides	3ul
ABE/CBE sgRNA expression plasmid	1ul
T4 DNA Ligase	1ul
10X T4 DNA ligase buffer	2ul
ddH_2_O	13ul

Place at 37 °C and ligate for 5 min (See Note 3)

4. According to the manufacturer’s protocol, transform 10 uL ligated product into 50 uL *E. coli* DH5a competent cells. The cells are plated onto an LB agar plate supplemented with the Ampicillin, and the plate is incubated at 37 °C for 14-16 h (See Note 4)
5. Randomly pick several colonies to verify the successful cloning by Sanger sequencing. Sequencing primer: ATTCTTTCCCCTGCACTGTACCCC (See Note 5)
6. Extract the plasmids by EndoFree Mini Plasmid Kit II according to the manufacturer’s protocol. Determine the concentration of the extracted plasmid using NanoDrop.

### Base Editing and Blasticidin Selection

7. Cells were plated into 48-well plates and transfected the next day at approximately 70% confluency.
8. 500 ng of epi-ABEmax/epi-AncBE4max plasmid was transfected using Lipofectamine 3000 according to the manufacturer’s instructions on day 1. (See note 6)
9. Cells were passaged on day 2 and selected by blasticidin. 2 μg/ml of blasticidin was added into the growth media, except on days 2, 6, and 11, where 8, 4, and 0 μg/ml of blasticidin were used, respectively. Transfected cells were harvested for analysis on days 6, 11, and 16. The antibiotic screening time can be shortened when editing efficiency is enough.
10. According to the manufacturer’s instructions, the genomic DNA was isolated using TIANamp Genomic DNA Kit. Targets of base editing were amplified by PCR using PrimeSTAR® Max DNA Polymerase. The PCR products were sequenced using Sanger sequencing, and the editing efficiency was analyzed by EditR (Kluesner et al., 2018) or BEAT (https://hanlab.cc/beat/).

### Single Cell-Derived Clone Screen

11. The antibiotic-iPSCs were passaged with EDTA. Then, 1 × 10^5^ cells were seeded on a Matrigel pre-coated 10 cm dish using mTeSR-1 cell culture medium with 5 μM of Y-27632. (See note 7)
12. Twenty-four hours later, the mTeSR-1 media was replaced by new media without Y-27632. This media was changed every two days.
13. Ten days after seeding, the single cell-derived clones were picked up using a 1 ml sterile syringe and divided into two halves. One half was placed on a Matrigel pre-coated 96-well plate for cell expansion. The other half extracted DNA using the TIANamp Genomic DNA Kit for DNA sequencing. (See note 8)

### Genotyping PCR

14. Design Forward and Reverse primers flanking the region targeted by the sgRNAs for the genotyping. The PCR will be performed to confirm the outcomes of base substitution. This experiment is to extract DNA from a small number of cells for PCR experiments, and the steps are as follows:

- Aspirate cells with a 50ul pipette, transfer to a 200ul PCR tube, and quick spin
- 95 °C for 10 min and cold down to 4 °C
- Add 2ul of 20 mg/ml proteinase K solution and mix by quick spin
- 55 °C for 1 h, 95 °C for 10 min and cold down to 4 °C

The solution obtained is the cellular DNA extraction solution, which can be used as a template for PCR.

**Table:** 

2x PrimeSTAR® Max DNA Polymerase	25 ul
Primer-F	2 ul
Primer-R	2 ul
DNA solution	8 ul
ddH_2_O	13 ul

- Gently mix all the reagents and collect them by a quick spin. The order of addition of the reagents can be random.
- Polymerase chain reaction (PCR)

(See note 9)

### Off-Target Analysis

15. For each target site, five potential off-targets were selected based on Cas-Offinder and PCR-amplified for Sanger sequencing.

### Cardiomyocyte Differentiation

16. Cells (∼90% confluency) were seeded on a Matrigel pre-coated 6-well plate at a ratio of 1:6 in mTeSR-1 media. (See note 10)
17. The media was changed to CDM3 supplemented with 6 μM of CHIR99021 when the cells reached ∼75% confluency.
18. After 48 h, the media was changed to CDM3 [7] supplemented with 2 μM of Wnt-C59. After 2 days, the media was changed to CDM3 and refreshed every 2 days. After differentiation for 7– 8 days, spontaneous contracting cells could be observed.
19. On day 12, Cardiomyocytes (CMs) were purified using a metabolic-selection method. The medium consisted of RPMI 1640 without glucose, 213 μg/ml of L-ascorbic acid 2-phosphate, 500 μg/ml of *Oryza sativa*-derived recombinant human albumin, and 5 mM of sodium DL-lactate.
20. After purification, CMs were cultured with RPMI 1640 and B27 (with insulin). For cellular maintenance, the medium was changed every 3 days.

### LQT Phenotype Identification Using MEA

21. CMs were digested with Accutase. (See note 11)
22. CytoView MEA24 plates (Axion Biosystems, Inc., Atlanta, United States) were pre-coated overnight using a 0.5% Matrigel phosphate-buffered saline (PBS) solution.
23. 15,000 CMs were plated on each multi-electrode array (MEA) well with RPMI/B27 medium and cultured for three days.
24. When the cellular electrophysiological activity became stable, the experimental data were recorded using Maestro EDGE (Axion Biosystems, Inc., Atlanta, United States) according to the MEA manual. The data were analyzed using the AxIS Navigator, Cardiac Analysis Tool, and IGOR software.

### Notes

1. The potential therapeutic targets were further screened according to the ABE/CBE sgRNA design rules, which must be in the form of 20 nt + PAM. The mutation site is in the sgRNA edit window 2-8. Therapeutic targets eligible for gene editing were obtained. To avoid bystander editing during gene editing, we recommend only one of the mutation loci in the edit window 2-8 of sgRNA. Other databases like ClinVar (https://www.ncbi.nlm.nih.gov/clinvar/)are also recommended.
2. **ttt** and **aac** are the sequences that matche the sticky end produced by the enzymatic cleavage of the plasmid used in this paper. You should add the appropriate sequence to the sticky end sequence produced by the plasmid you are using.
3. The sgRNA expression plasmids in this protocol contain epi-ABEmax, epi-AncBE4max, which is an all-in-one episomal vector expressing a single guide RNA (sgRNA) with an adenine base editor (ABE) or a cytosine base editor (CBE). If you are using two plasmids for sgRNA and base editor expressing, you only need to ligate oligonucleotides to the sgRNA expression vector.
4. You should choose the appropriate antibiotic according to the resistance expressed by your plasmid.
5. You should choose the appropriate sequencing primer according to your plasmid.
6. We recommend the use of the LONZA 4D Nuclear Transfection System to transfer plasmids into cells, programmed as CA137.
7. EDTA is less toxic to cells. To obtain single cells, we try to increase the contact time between EDTA and the cells until the cells are observed as single cells under the microscope. Typically contact time is10-20 min.
8. The cell clone must not be too small, or the clone will fail to pick up. Generally, under a 10x microscope, the cell clone should fulfill the entire field. The microscope should be transferred to a biosafety cabinet in advance and UV irradiated for at least half an hour.
9. PCR conditions should be referred to the instructions for the polymerase used. We should determine primer Tm values in advance. The amount of DNA extracted by our method is minimal, and therefore the number of cycles of PCR should be increased, with a recommended setting of 38 cycles.
10. Matrigel was diluted using pre-cooled PBS solution at a ratio of 1:500. The process of CM differentiation is susceptible to mycoplasma, and we strongly recommend testing the mycoplasma before CM differentiation. The cell culture medium supernatant is aspirated and used as a template for PCR amplification using mycoplasma-specific primers and agarose gels for validation. Mycoplasma-specific primer [27]-F: CACCATCTGTCACTCTGTTAAC, R: GGAGCAAACAGGATTAGATAC
11. If the CM differentiation is inefficient, dead cells are produced, and many cell fragments are released during CM purification. These cell fragments can bind to collagen, the cardiomyocyte, in a pile and hinder the CM digestion. Collagenase can be an excellent solution to this problem. Add collagenase solution to CM and incubate at 37 °C for 1 h. Aspirate the collagenase solution, add Accutase, and placed at 37 °C for 30 min.
12. Summary of base editing efficiency

**Table:** 

Gene	mutation	Total number of clones	Heterozygous clones	Homozygous clones
KCNQ1	L114P	52	26(50%)	3(5.8%)
KCNQ1	R190Q	77	22(28.6%)	6(7.8%)
KCNH2	Y616C	4	4(100%)	0(0)
KCNH2	Y475C	41	18(40.9%)	8(19.5%
SCN5A	E1784K	107	32(29.9%)	5(4.7%)
SCN5A	R1879W	47	2(4.3%)	8(17%)
Average			31.7%	9.1%

## Data Availability

Not applicable.
